# An Organic Khorasan Wheat-Based Replacement Diet Improves Risk Profile of Patients with Acute Coronary Syndrome: A Randomized Crossover Trial

**DOI:** 10.3390/nu7053401

**Published:** 2015-05-11

**Authors:** Anne Whittaker, Francesco Sofi, Maria Luisa Eliana Luisi, Elena Rafanelli, Claudia Fiorillo, Matteo Becatti, Rosanna Abbate, Alessandro Casini, Gian Franco Gensini, Stefano Benedettelli

**Affiliations:** 1 Department of Agrifood Production and Environmental Sciences, University of Florence, Piazzale delle Cascine 18, 50144 Florence, Italy; E-Mails: anne.whittaker.anne@gmail.com (A.W.); stefano.benedettelli@unifi.it (S.B.); 2 Unit of Clinical Nutrition, Careggi University Hospital, Largo Brambilla 3, 50134 Florence, Italy; E-Mail: alessandro.casini@unifi.it; 3 Department of Experimental and Clinical Medicine, University of Florence, Largo Brambilla 3, 50134, Florence, Italy; E-Mails: rosanna.bbate@unifi.it (R.A.); gianfranco.gensini@unifi.it (G.F.G.); 4 Don Carlo Gnocchi Foundation Florence, Via di Scandicci 269, 50143, Florence, Italy; E-Mails: mleluisi@dongnocchi.it (M.L.L.); elena.rafanelli@dongnocchi.it (E.R.); 5 Department of Clinical and Experimental Biomedical Sciences, Interdipartimental Center for Research on Food and Nutrition, University of Florence, Sesto Fiorentino, Florence, 50019, Italy; E-Mails: claudia.fiorillo@unifi.it (C.F.); matteo.becatti@unifi.it (M.B.)

**Keywords:** khorasan wheat, conventional wheat, acute coronary syndrome, secondary prevention, diet

## Abstract

Khorasan wheat is an ancient grain with previously reported health benefits in clinically healthy subjects. The aim of this study was to examine whether a replacement diet, thereby substituting all other cereal grains, with products made with organic khorasan wheat could provide additive protective effects in reducing lipid, oxidative and inflammatory risk factors, in patients with Acute Coronary Syndromes (ACS) in comparison to a similar replacement diet using products made from organic modern wheat. A randomized double-blinded crossover trial with two intervention phases was conducted on 22 ACS patients (9 F; 13 M). The patients were assigned to consume products (bread, pasta, biscuits and crackers) made either from organic semi-whole khorasan wheat or organic semi-whole control wheat for eight weeks in a random order. On average, patients ingested 62.0 g dry weight (DW) day^−1^ khorasan or control semolina; and 140.5 g DW day^−1^ khorasan or control flour, respectively. An eight-week washout period was implemented between the respective interventions. Blood analyses were performed both at the beginning and end of each intervention phase; thereby permitting a comparison of both the khorasan and control intervention phases, respectively, on circulatory risk factors for the same patient. Consumption of products made with khorasan wheat resulted in a significant amelioration in total cholesterol (−6.8%), low-density lipoprotein cholesterol (LDL-C) (−8.1%) glucose (−8%) and insulin (−24.6%) from baseline levels, independently of age, sex, traditional risk factors, medication and diet quality. Moreover, there was a significant reduction in reactive oxygen species (ROS), lipoperoxidation of circulating monocytes and lymphocytes, as well as in the levels of Tumor Necrosis Factor-alpha. No significant differences from baseline in the same patients were observed after the conventional control wheat intervention phase. The present results suggest that a replacement diet with cereal products made from organic khorasan wheat provides additional protection in patients with ACS. Circulating cardiovascular risk factors, including lipid parameters, and markers of both oxidative stress and inflammatory status, were reduced, irrespective of the number and combination of medicinal therapies with proven efficacy in secondary prevention.

## 1. Introduction

Cardiovascular Disease (CVD) causes approximately one-third of all deaths globally in both developed and developing countries. Coronary Heart Disease is the largest contributor of CVD, and encompasses Acute Coronary Syndrome (ACS), which is an acute pathology associated with atherosclerotic plaque rupture and interruption of coronary blood to myocardial tissue. Patients with ACS are at particularly high risk of both fatal and non-fatal recurrent cardiovascular events despite stringent medical therapies [1]. Given the global impact of CVD, much research has focused on the beneficial effects of easily assessable modifiable risk factors, such as nutritional factors [2].

Numerous large cohort studies have shown that a healthy diet (in particular, adherence to a Mediterranean diet) provides significant protection against myocardial infarction (MI) and CVD-related mortality in participants without prior established CVD [2,3,4]. Although fewer studies have been conducted on patients in secondary prevention, higher adherence to a healthy diet has been shown to reduce the incidence of mortality in CVD patients [2,4,5]. In particular, the prospective cohort study of Dehghan *et al.* [5], involving 31,546 individuals over a 56-month follow-up period, showed the additive protective impact of healthy diet on recurrent cardiovascular events in patients dependent on drug therapy. Given the established benefits of the Mediterranean diet (of interest to the present study based in Italy), we were particularly interested in focusing on cereal products, which form the basis of the Mediterranean dietary pyramid. In particular, there is currently a renewed interest in old varieties (defined as varieties in existence prior to intensive selection for gluten quantity and quality) of both *Triticum aestivum* and *Triticum durum*, which represent a valuable source of biodiversity in functional components. The health benefits conferred, were suggested to be attributable to anti-oxidant and anti-inflammatory compounds [6,7,8].

Of the more ancient varieties, *Triticum turgidum* subsp *turanicum*, also known as khorasan wheat is emerging as an alternative health grain. In our previous study [9], a replacement diet with organic khorasan wheat pasta and bread products was shown to exercise a beneficial effect on cardiovascular risk factors (total cholesterol, low-density lipoprotein cholesterol (LDL-C), blood glucose, total antioxidant capacity, and various pro-inflammatory cytokines) in a healthy population with no prior clinical manifestations of CVD. No such benefits were observed from a replacement diet using organic conventional (modern or commercial varieties) of Italian *T. durum* (pasta) and *T. aestivum* (bread) wheat. Despite benefits in reducing cardiovascular risk in a healthy population, the question remains as to whether additive protection could be provided in patients already dependent on drug therapy for secondary cardiovascular prevention. Therefore, the objective of the present investigation was to assess the potential effects of an organic khorasan wheat replacement diet on reducing various circulatory biochemical, oxidative stress-related and inflammatory risk factors in patients with established ACS on drug therapy. As a control, conventional wheat, cultivated in organic agriculture and transformed in the same manner, was also included.

## 2. Methods and Materials

### 2.1. Study Population

The study population was initially comprised of 23 patients, one of whom refused to participate before the onset of the study. The final study population was, therefore, comprised of 22 patients (13 men; 9 women) with a diagnosis of ACS. The median age was 61 (47–75 year age range), with a mean body mass index (BMI = kg m^−2^) of 26.9 ± 4.4. All patients were recruited on entry to the clinic following consultation at the Department of Experimental and Clinical Medicine, University of Florence and/or at the Foundation Don Carlo Gnocchi, Onlus IRCCS, Florence, Italy.

Diagnosis of ACS was determined according to the following criteria: (a) typical chest pain lasting >30 min in the previous 24 h by the first medical observation; (b) rise in serum CK-MB (Creatin Kinase Myoglobin) or CK at least twice above the normal upper limit within 72 h of symptom onset and/or rise in serum Troponin I or T above the normal upper limit; and (c) evidence of ACS on the ECG (Electrocardiogram, ST-segment elevation ≥0.1 mV in two or more adjacent leads; new onset left bundle-branch block; ST-segment depression of ≥1 mm in two or more adjacent leads; new T waves inversion ≥ 1 mm in two or more adjacent leads; pseudo normalization T waves). Patients were eligible for enrollment if they presented criteria (a) and at least one of the (b) or (c) criteria. However, the inclusion criteria for patient participation in the present study necessitated that ACS patients were required to: be of an age ranging between 20 and 80 years, have neither liver nor renal failure, have neither a gluten allergy nor gastrointestinal disorders (e.g., chronic constipation, diarrhea, inflammatory bowel disease, irritable bowel syndrome, or other chronic gastrointestinal complaints) and gall bladder problems. Volunteers were instructed not to alter their usual dietary or fluid intakes.

Written informed consent was obtained from each patient before the initial screening visit and before the initiation of the experimental trial. The institutional review board at the University of Florence approved the study protocol.

### 2.2. Data Collection and Measurement

Patients were interviewed and examined at Careggi hospital (Florence) through the use of standardized methods. Information about personal medical history, demographics, family history of coronary or other atherosclerotic diseases, medication and lifestyle habits (related to smoking habit, diet, physical exercise, weight, blood pressure, lipids and diabetes) was obtained at the time of the interview. All information relating to the above-mentioned aspects served as descriptive supplementary information pertaining to the current study population, but was not used as a basis for exclusion criteria. Physicians using standardized protocols conducted a physical examination, blood pressure measurements, laboratory tests and a dietary survey. Body mass index (BMI) was calculated as weight (kg) height (m^−2^): Patients were classified overweight if their BMI was more than 25 kg m^−2^ but less than 30 kg m^−2^, and obese if their BMI was 30 kg m^−2^ or more. Hospital attendants measured blood pressure on the patient’s right upper arm in a sitting position. Raised blood pressure was defined as systolic blood pressure 140 mmHg or more and/or diastolic blood pressure 90 mmHg or more for primary prevention and as systolic blood pressure 130 mmHg or more and/or diastolic blood pressure 80 mmHg or more for secondary prevention according to the guidelines of the European Society of Cardiology. Smokers were defined as those who smoked at the time of physical examination. Diabetic subjects were defined in agreement with the American Diabetes Association or on the basis of self-reported data (if confirmed by medication or chart review). Dyslipidaemia was defined according to the Third report of the National Cholesterol Education Program (NCEP-III) or if they reported taking antidyslipidemic drugs, as verified by the physician. Physical activity was assessed as either absent (sedentary lifestyle), mild or moderate based on the duration and intensity of physical activity over the preceding 6 months. Baseline adherence to a Mediterranean diet was evaluated from a questionnaire that included 3 categories of consumption (min 0 points, max 2 points) for each food group (cereals, fruit, vegetables, legumes, olive oil, meat products, dairy products, fish and alcohol) composing the Mediterranean diet. The questionnaire permitted the assignment of an overall score (min 0 points, max 18 points) to each patient, thereby ranking the degree of adherence [3].

### 2.3. Wheat Varieties

Organically grown khorasan wheat (*Tritucum turgidum* subsp *turanicum*), provided by Kamut Enterprise of Europe (KEE), Belgium (Kamut^®^ is a registered trademark of Kamut International Ltd and Kamut Enterprises of Europe bvba) and obtained from Saskatchewan, Canada, the principle cultivation area, was utilized as the ancient wheat under investigation. Different countries utilize different terminology to classify the different types of milled flour (obtained from soft wheat for the preparation of bread and biscuits) and semolina (obtained from durum wheat for the preparation of pasta). For this reason, the ash content, which is positively correlated to extraction rate, is reported for comparative purposes when dealing with different classification systems. Italian flour and semolina are classified according to the ash content (mineral content), with a higher ash content being representative of a higher content of bran particles after milling.

Khorasan semolina (ash content, 1.10%–1.35%) and flour (ash content, 1.0%) were processed by *Molino SIMA S.C.A.R.L.* (Argenta, Ferrara, Italy). For the organic khorasan wheat, different milling procedures were employed to produce the granulated semolina (analogous to semi-whole wheat semolina) and flour (analogous to semi-whole wheat flour), respectively, thereby resulting in differences in ash content. As the control (from here on referred to as the control wheat), semi-whole-wheat granulated semolina (ash content 1.0%–1.35%) and semi-whole wheat flour (ash content, 0.95%), derived from a mix of organically-cultivated commercial Italian durum (*T. durum*) varieties and soft wheat (*T. aestivum*) varieties, respectively were processed by the same mill. Hence, for the control, the differences in ash content between the granulated semolina and the flour, respectively, were attributable not only to differences in milling procedures, but also to species.

All transformation preparation procedures were identical for both the khorasan wheat and modern control wheat under study. Pasta was prepared from both the khorasan wheat and control semolina according to the artisan manufacturing procedures performed by the *Pastificio Artigiano FABBRI s.a.s.* (Strada in Chianti, Firenze, Italy). The pasta was made from the respective semolina sources, and contained no additives. The bread, biscuits and crackers were made by the artisan enterprise of *Panificio Menchetti Pietro di Santi e Figli s.n.c.* (Cesa Marciano della Chiana, Arezzo, Italy) using the khorasan wheat and control flour. Naturally, leavened sourdough bread was prepared, without salt according traditional Tuscan style. Besides the flour composition, dry crackers contained 20% extra-virgin olive oil, whereas the biscuits contained 20% sugar and 10% butter, and one egg per 100 g flour.

### 2.4. Study Design

The study was a randomized, double-blinded, crossover trial designed to test whether a replacement diet with organic khorasan wheat products could provide additive benefits to ACS patients in secondary prevention, in comparison to a similar replacement diet using modern control wheat products. By replacement diet it is intended that all other cereal grains were to be substituted by the khorasan and control wheat, respectively. Moreover, subjects were not permitted to eat other grain products during the respective intervention phases. Patients were informed that both the modern control and khorasan wheat were potentially beneficial, and that they were organic and prepared by artisan methods. All products were packaged with no labels attached to the packages. The patients were randomly divided into two groups (11 individuals group^−1^), and a crossover study design with two intervention phases was implemented. Each group was, respectively, assigned to consume either the khorasan or the control wheat products. The first intervention phase was initiated in mid-October 2013, with participants in both groups receiving 500 g week^−1^ of pasta, 1000 g day^−1^ of bread, 250 g week^−1^ of crackers and 250 g week^−1^ of biscuits for a period of 8 weeks. The average daily intake of both khorasan and control semolina was 62.0 g DW, whereas the average daily intake of both khorasan and control flour (from all the products consumed) amounted to 140.5 g DW. Both khorasan and control wheat products were given to each subject every week; after having controlled that, subjects ate the products given. A washout period of 8 weeks was then effected, during which patients were permitted to eat all foods according to their “normal” dietary habits. The second intervention phase of 8 weeks was initiated in mid-February 2014, and each group crossed-over to respectively consume either the khorasan or control wheat products, not consumed during the first intervention phase. At baseline and after each intervention, all subjects were examined between 7:00 a.m. and 9:30 a.m. after an overnight fasting period. Furthermore, subjects were asked not to engage in strenuous physical activity during the day before the examination.

### 2.5. Characteristics of the Wheat Varieties

The secondary metabolite content and mineral element content were measured as previously described [9].

### 2.6. Blood Biochemical and Inflammatory Profiles

Venous blood samples were collected into evacuated plastic tubes (Vacutainer). Samples, obtained by centrifuging at 3000 g for 15 min at 4 °C, were stored in aliquots at −80 °C until analysis. Lipid variables, blood glucose, insulin, and serum electrolytes were assessed by conventional methods. Pro- and anti-inflammatory cytokines were determined by using the Bio-Plex cytokine assay (Bio-Rad Laboratories Inc., Hercules, CA, USA), according to the manufacturer’s instructions.

### 2.7. Assessment of Reactive Oxygen Species’ Production and Lipoperoxidation

After collection, 2 mL of BD FACS Lysing Solution (Becton Dickinson Biosciences, San Jose, CA, USA) was added to 100 μL EDTA-anticoagulated blood samples, gently mixed, and incubated at room temperature in the dark for 10 min, according to the manufacturer’s protocol. Thereafter, the cells were centrifuged, the supernatant discarded, and cells washed twice in PBS. Leukocyte reactive oxygen species (ROS) generation and lipoperoxidation was measured according to Becatti *et al.* [10]. Cells were incubated with *H_2_DCFDA* (2.5 μM) (Invitrogen, CA, USA) and BODIPY 581/591 C11 (5 μM) (Invitrogen, CA, USA) in RPMI without serum and phenol red for 15 min at 37 °C, respectively. After labeling, cells were washed, resuspended in PBS, and analyzed immediately using a FACSCanto flow cytometer (Becton-Dickinson, San Jose, CA, USA). The sample flow rate was adjusted to about 1000 cells s^−1^. For a single analysis, the fluorescence properties of 20,000 leukocytes were collected. The respective gates were defined using the distinctive forward-scatter and side-scatter properties of the individual cell populations. Moreover, cell viability was controlled by flow cytometry with propidium iodide staining and was shown to exceed 95%. Data was analyzed using the BD FACSDiva software (Becton-Dickinson, San Jose, CA, USA).

### 2.8. Thiobarbituric Acid Reactive Substance Assay and Total Antioxidant Capacity

Malondialdehyde (MDA) is the last product of fatty acid peroxidation. Plasma levels of MDA were quantified using the thiobarbituric acid reactive substance (TBARS) assay kit (Oxitek-ZeptoMetrix Corporation Buffalo, NY, USA) as previously reported [9,10]. Similarly, Total Antioxidant Capacity (TAC), accounting for total hydrophilic ROS scavengers, was measured in plasma by a chemiluminescence assay using the photoprotein Pholasin (Abel Antioxidant Test Kit, Knight Scientific Ltd, Plymouth, UK) as reported previously in Sofi *et al.* [9].

### 2.9. Statistical Analysis

Statistical analysis was performed by using the statistical package PASW 18.0 for Macintosh (SPSS Inc., Chicago, IL). Results are expressed either as mean ± SD or as median and range, as considered appropriate. One-way ANOVA was used for testing differences between khorasan and control flour and semolina. The analyses were simplified by calculating the absolute change for each variable tested (mean value at baseline subtracted from the mean value after intervention for each subject) with independent *t* sample tests. No carryover effect was observed. Therefore, all data were treated as paired samples from a crossover study. Data that were not normally distributed were logarithmically transformed. The two interventions were analyzed by taking into account both phases in the two groups of subjects at different stages. Data were analyzed by using paired *t* tests for significant differences between changes observed during test and control intervention periods. Moreover, in order to compare the effect of khorasan products *versus* baseline and *vs.* the control products, a general linear model for repeated measurements, after adjustment for age and gender, modifiable risk factors (smoking habit, sedentary lifestyle, hypertension, dyslipidaemia, diabetes) and medication was performed. A value of *p* < 0.05 was considered to indicate statistical significance.

## 3. Results

### 3.1. Characteristics of the Wheat Varieties

Flour and semolina were characterized for total polyphenol, flavonoid, carotenoid and anti-radical activity, expressed as anti-radical power (ARP). The major differences reported in the above-mentioned constituents were between the khorasan wheat and control wheat flour, or the part of the diet involving the consumption of bread, biscuits and crackers (Table 1). A significantly higher antioxidant content (polyphenols, flavonoids and carotenoids) as well as ARP and 2,2-dyphenyl-1-picrylhydrazyl (DPPH) antiradical activity was apparent in the khorasan wheat flour with respect to the control wheat flour. With the exception of the carotenoid content in the khorasan wheat semolina, no differences in polyphenol and flavonoid content or ARP were evident between the khorasan wheat and control wheat semolina. Flour and semolina were also characterized for various mineral elements. Both khorasan wheat semolina and flour contained significantly higher selenium contents. Vanadium was higher in khorasan wheat than in the control wheat flour (Table 1). Given that total soluble and insoluble fiber and total starch contents did not vary between the khorasan and control wheat [9], these parameters were not examined but reference can be made to our previous study [9].

### 3.2. Study Population Characteristics

Baseline demographic, clinical, and laboratory parameters were not significantly different between the two groups assigned to initiate the experimental trial by consuming either the khorasan or modern control wheat in the first intervention phase (data not reported). The study population was comprised of individuals at cardiovascular risk with an overall 45.5, 40.9, 59.1, 50.0 and 13.6% of patients reporting positive for smoking habit, sedentary lifestyle, hypertension, dyslipidemia and diabetes, respectively. Behavioral risk factors related to smoking habit and sedentary lifestyle were unmodified during the course of the study, whereas the remaining modifiable risk factors were maintained under control by medicinal therapies, which were not altered during the course of the trial. Of the 11 different preventative drugs in use by the study population, 100, 81.8 and 50% of the patients were on statins, beta-blockers and aspirins, respectively. Two-thirds of the participants (16 patients) were taking between four and seven different drugs. At baseline, it was shown that on average, the patients adhered relatively well to a Mediterranean diet (mean score: 12.6). Given the interest in the cereal component of the Mediterranean diet, which forms the basis of the diet, it was noteworthy that 68.2% and 31.8% of the patients had reported a cereal consumption of 1.5 and 1–1.5 portions day^−1^ at baseline. Of the cereals, wheat is the staple grain consumed in Italy, and is consumed predominantly in the form of both pasta and bread at meals, and for many in the form of biscuits for breakfast. Smaller portions in the form of crackers and biscuits are also consumed during coffee breaks. During the trial, the participants consumed these portions, substituting other wheat products normally eaten with the products provided. There was no increase in BMI reported at the end of the trial, indicating that the patients were not consuming an excess of wheat products.

**Table 1:** Composition of khorasan wheat and control wheat.

Variable	Khorasan Wheat (Semolina)	Control wheat (Semolina)	*p*	Khorasan Wheat (Flour)	Control Wheat (Flour)	*p*
**Protein**, %	14.6 ± 0.07	13.3 ± 0.11	0.3	14.7 ± 0.06	13.9 ± 0.09	0.3
**ARP**	6.06 ± 0.41	5.80 ± 0.54	0.8	6.04 ± 0.33	5.08 ± 0.16	0.03
**Polyphenols**, mg g^−1^DM	1.23 ± 0.04	1.29 ± 0.06	0.4	1.05 ± 0.04	0.98 ± 0.03	0.03
**Flavonoids**, mg g^−1^DM	0.23 ± 0.02	0.24 ± 0.03	0.5	0.20 ± 0.03	0.11 ± 0.07	0.001
**Carotenoids**, mg g^−1^DM	18.1 ± 1.61	15.3 ± 0.28	0.03	15.3 ± 0.21	5.81 ± 0.06	0.02
**Vanadium**, mg kg^−1^	1.18 ± 0.08	0.81 ± 0.20	0.3	1.16 ± 0.09	0.48 ± 0.20	0.04
**Iron**, mg kg^−1^	22.9 ± 1.27	23.6 ± 0.38	0.2	21.4 ± 1.41	13.6 ± 3.15	0.08
**Potassium**, mg kg^−1^	1819 ± 25.5	1989 ± 346.5	0.5	1732.5 ± 81.3	1221.5 ± 474.5	0.7
**Magnesium**, mg kg^−1^	1044.2 ± 70.4	915.4 ± 89.4	0.3	978.1 ± 0.35	672.2 ± 234.9	0.2
**Phosphorus**, mg kg^−1^	2114.5 ± 71.4	1925.5 ± 303.3	0.1	2045 ± 91.9	1422.5 ± 391	0.3
**Selenium**, mg kg^−1^	1.82 ± 0.15	1.08 ± 0.39	0.02	1.19 ± 0.14	0.50 ± 0.11	0.04
**Zinc**, mg kg^−1^	21.1 ± 0.22	21.5 ± 0.92	0.1	19.4 ± 1.11	13.6 ± 5.04	0.6

Data are reported as mean ± SD; One-way ANOVA test.

### 3.3. Modifications in the Biochemical Profile

All biochemical, redox and inflammatory variables, measured before and after each respective replacement diet, were adjusted for confounding baseline demographic and traditional CVD risk factors, as well as for secondary prevention medicines. After the dietary replacement with khorasan wheat products, patients experienced a significant amelioration in blood glucose, insulin, total cholesterol and LDL-cholesterol, whereas no changes during the phase of intervention with the control wheat products were reported (Table 2). In particular, total cholesterol significantly reduced of 6.8%, LDL-cholesterol of 8.1%, glucose of 8% and insulin of 24.6%. A significant increase in magnesium was also reported only after the khorasan wheat dietary intervention (Table 2). All remaining parameters remained unchanged.

### 3.4. Modifications in the Redox Status

With the aim of evaluating antioxidant potential, blood redox status before and after each intervention phase was tested (Table 3). ROS production and lipoperoxidation in circulating monocytes and lymphocytes was significantly decreased after the khorasan wheat replacement diet. This effect was not evident after the consumption of control wheat products. ROS production and lipoperoxidation of granulocytes and plasma levels of MDA and TAC remained unchanged after both the khorasan wheat and control wheat dietary interventions (Table 3).

### 3.5. Modifications in the inflammatory profile

In addition, the inflammatory profile was also tested in the study population through an evaluation of various pro- and anti-inflammatory cytokines (Table 4). A significant reduction in the circulating levels of pro-inflammatory Tumor Necrosis Factor (TNF)-alpha (−34.5%) was observed after the period of dietary replacement with khorasan wheat products. This change was not evident after the consumption of the control wheat products. No significant changes were reported for the remaining pro- and anti-inflammatory cytokines.

## 4. Discussion

Investigation of the potential effects of organic khorasan wheat on patients with ACS was based on our previous study showing that a replacement diet was effective in reducing cardiovascular risk factors on a healthy population with no prior clinical manifestations of CVD [9]. To our knowledge, the present work is the first to evaluate the functional efficacy of this wheat in a study population with a chronic disease, independently from drug therapy. Statins, beta-blockers and antiplatelet agents (aspirin) were taken, either singularly or collectively, by all patients to reduce cardiovascular risk as well as modifiable CVD risk factors such as blood pressure/hypertension, cholesterol/dyslipidemia, as well as type 2 diabetes mellitus. Given that drug therapy was maintained constant throughout the trial, and no lifestyle changes were implemented to reduce additional major modifiable cardiovascular risk factors, such as smoking habit and sedentary lifestyle, the study permitted the evaluation of beneficial changes attributable to replacement diet with either the khorasan or the conventional control wheat. Notwithstanding stringent medicinal therapy, the present work is a novel contribution showing that a replacement diet with organic khorasan wheat impacted positively by further down-regulating several key markers associated with recurrent cardiovascular accident, including total cholesterol, LDL-cholesterol (LDL-C), blood glucose, ROS production and lipoperoxidation of leukocytes (monocytes and lymphocytes) and TNF-alpha. These effects were not observed after consumption of the commercial modern wheat control.

As outlined previously [9], differences in protective effects were not attributable to the fact that khorasan wheat was organic semi-whole wheat, since the same patients also consumed an organic, semi-whole control wheat. The present work suggests that khorasan wheat possesses health-promoting properties not evident in commercial conventional varieties. Given that antioxidant supplement studies have provided inconclusive results as to the benefits of many antioxidants, it is of interest that the intake of health-promoting properties can be accomplished in the form of a staple food choice. This provides a healthy means, not only in reducing the risk of chronic disease development but also towards combatting the already established presence of chronic disease. Although the present study was conducted on organic khorasan wheat, it raises an interesting question as to whether similar benefits may be attained from other ancient grains, as well as older varieties of *T. durum* and *T. aestivum* as preliminary studies have suggested [8]. Research in this area is warranting, based on the preliminary findings reported.

The consumption of organic khorasan wheat products resulted in a significant decrease in both total cholesterol and LDL-C. Baseline levels of both total cholesterol and LDL-C were significantly lower in the present study than those observed for a healthy population [9]. Lipid-lowering therapy is imperative as LDL-C, a well-established biomarker of recurrent risk, is subject to oxidation by ROS. A significant decrease in ROS production and lipoperoxidation of circulating monocytes and lymphocytes after a replacement diet with organic khorasan wheat products was also evident. The decrease was particularly significant for the monocytes, the predominant leukocyte contributor in the atherosclerotic plaque formation [11]. Improvements in the redox status was independent of the pleiotropic effects of statin therapy, which have been suggested to include reducing ROS production and susceptibility of LDL-C oxidation, as well as decreasing leukocyte count and associated activities [10,11]. However, reductions in ROS production and stress-induced inflammatory effects are suggested to be the result of multiple compounds with differential effects, best derived from valuable food sources [12,13].

In this study, pro-inflammatory TNF-alpha levels decreased significantly, only after consumption of the organic khorasan wheat products. Expressed in macrophages and lymphocytes throughout atherosclerotic plaque formation, TNF-alpha has been termed the “master inflammatory cytokine” acting as a potent protagonist in the induction of pro-inflammatory gene expression [11]. Moreover, TNF-alpha stimulates ROS production and is considered a principle effector of plaque instability [11,14]. Elevated levels are associated with an increased risk of recurrent coronary events after MI [15]. Despite the fact that beta-blockers are suggested to attenuate TNF-alpha [16], an additive effect ascribed to organic khorasan wheat was also evident in this study.

Despite the increased selenium contents in khorasan, in our previous study [9], redox and inflammatory improvements associated with the consumption of khorasan wheat were reported notwithstanding the fact that selenium contents in the control were not significantly different. This suggests that the benefits derived are attributable, not to single components in isolation, but to potential synergistic effects of numerous components [17]. Research in recent years has shown compelling evidence that various secondary metabolites (traditionally viewed exclusively as antioxidants) appear to exercise effect through involvement in signaling pathways [13,14]. Various polyphenol molecules that have been implicated in cell signaling pathways include the oxygen-stress-sensitive nuclear factor (NF-κB), which mediates TNF-alpha signaling [13,14]. Hence, it is likely that benefits exerted by organic khorasan wheat may reside not in the overall quantity of secondary metabolites (which in wheat as a food source are relatively low, and which were also not largely different between khorasan and the conventional wheat). Rather positive effects may reside in the existence of novel secondary metabolite molecules or specific polyphenol isoforms, as has been shown for other old wheat varieties [6,7], that may contain signaling capacities not evident in the conventional varieties. This is warranting of investigation.

**Table 2:** Modifications in biochemical parameters ^a^.

Variable	Khorasan Pre	Khorasan Post	Control Pre	Control Post	Change, Khorasan	Change, Control	*p*
**Blood glucose**, mmol L^−1^	5.55 (5.14–5.96)	5.11 (4.85–5.37) *	5.29 (4.95–5.65)	5.38 (5.08–5.69)	−0.44 (−0.76; −0.13)	0.09 (−0.1; 0.27)	0.03
**Insulin**, pmol L^−1^	101.1 (70.2–132)	76.2 (53.2–99.1) *	94.9 (70.2–119.7)	90.9 (64.9–117.1)	−24.9 (−41.6; −8.3)	−4.0 (−18.3; 10.3)	0.006
**Total cholesterol**, mmol L^−1^	4.43 (3.96–4.90)	4.13 (3.71–4.55) *	4.36 (3.89–4.82)	4.49 (4.03–4.94)	−0.30 (−0.45; −0.15)	0.13 (−0.07; 0.34)	0.001
**LDL-Cholesterol**, mmol L^−1^	2.29 (1.96–2.64)	2.11 (1.81–2.41) *	2.32 (1.97–2.67)	2.36 (2.02–2.70)	−0.18 (−0.28; −0.09)	0.04 (−0.09; −0.17)	0.001
**HDL-Cholesterol**, mmol L^−1^	1.42 (1.24–1.61)	1.39 (1.23–1.54)	1.45 (1.26–1.64)	1.41 (1.24–1.58)	−0.03 (−0.12; 0.05)	−0.04 (−0.13; 0.06)	0.4
**Triglycerides**, mmol L^−1^	1.39 (1.17–1.61)	1.33 (1.10–1.55)	1.29 (1.05–1.52)	1.37 (1.16–1.57)	−0.06 (−0.14; 0.01)	0.08 (−0.06; 0.23)	0.2
**Sodium**, mmol L^−1^	140.3 (139.4–141.1)	139.9 (139–140.8)	139.8 (138.9–140.6)	139.9 (139.1–140.7)	−0.4 (−1.1; 0.4)	0.1 (−0.7; 0.9)	0.8
**Potassium**, mmol L^−1^	4.34 (4.17–4.51)	4.44 (4.26–4.62)	4.31 (4.13–4.48)	4.26 (4.09–4.43)	0.10 (−0.08; 0.3)	−0.05 (−0.2; 0.08)	0.5
**Magnesium**, mmol L^−1^	0.87 (0.84–0.89)	0.89 (0.87–0.91) *	0.90 (0.86–0.94)	0.80 (0.68–0.92)	0.02 (0.002; 0.5)	−0.10 (−0.22; 0.02)	0.04
**Phosporus**, mmol L^−1^	1.09 (1.03–1.15)	1.12 (1.04–1.19)	1.12 (1.06–1.18)	1.12 (1.05–1.18)	0.03 (−0.07; 0.14)	0 (−0.04; 0.04)	0.9
**Iron**, μmol L^−1^	15 (12.6–17.4)	16.1 (13.4–18.8)	15.6 (12.6–18.7)	17.1 (13.6–20.5)	1.1 (−1.1; 3.3)	1.5 (−1.8; 4.7)	0.3

Data are reported as geometric mean and (range). General linear model adjusted for age, gender, number of medications, adherence score to MDLDL: Low-Density Lipoprotein; HDL: High-Density Lipoprotein. * *p* < 0.05 for comparison between pre- and post-intervention values (paired *t*-test). ^a^ Comparison between absolute changes induced by the two interventions (General linear model for repeated measurements).

**Table 3:** Modifications in oxidative stress—related parameters ^a^.

Variable	Khorasan Pre	Khorasan Post	Control Pre	Control Post	Change, Khorasan	Change, Control	*p*
**L-derived ROS**, RFU	1432 (1302.2–1561.9)	1274 (1149.3–1398.8) *	1355.2 (1200.5–1509.9)	1395 (1241.2–1548.8)	−158 (−306.1; −9.8)	39.8 (−94.5; 174.1)	0.003
**M-derived ROS**, RFU	2671.9 (2154.9–2925.6)	2317.6 (2113.7–2521.5) *	2642.9 (2154.9–2770.9)	2595.9 (2265.6–2926.3)	−354.2 (−568.9; −139.6)	133.1 (−3.9; −270.1)	0.002
**G-derived ROS**, RFU	3177.1 (2851.9–3502.3)	3228.7 (2808.8–3648.7)	3760.7 (3159.5–4361.9)	3386.1 (2956.8–3815.4)	51.6 (-292.7; 395.9)	−374.6 (−922.8; 180.6)	0.2
**TAC**, μmol mL^−1^	8.06 (7.66–8.46)	8.37 (7.85–8.89)	7.88 (7.44–8.32)	7.84 (7.39–8.28)	0.31 (−0.09; 0.7)	−0.04 (−0.6; 0.5)	0.9
**MDA**, nmol mL^−1^	6.94 (5.04–8.83)	6.89 (5.15–8.63)	7.49 (5.37–9.61)	6.44 (4.4–8.48)	−0.05 (−0.8; 0.7)	−1.05 (−2.1; 0.02)	0.06
L-lipoperox, RFU	2105.6 (1868.9–2342.2)	1779.3 (1544.1–2014.5) *	2215 (1876.7–2553.4)	1908.2 (1690.2–2126.2)	−326.3 (-601.4; −51.1)	−306.9 (−677.4; 63.7)	0.004
M-lipoperox, RFU	4869.7 (4273.3–5466.1)	4244.1 (3830.4–4657.8) *	4926.9 (4114.2–5739.7)	4977.8 (4386.6–5569.1)	−625.5 (−1097.5; −153.6)	50.9 (−796; 897.7)	0.001
G-lipoperox, RFU	6892.5 (6240.9–7544)	7049.3 (6232.8–7864.8)	8025.3 (6733.2–9317.3)	7518.5 (6635.3–8401.6)	156.8 (−589.4; 903.1)	−506.8 (−1705.8; 692.1)	0.4

L = lymphocytes; M = monocytes; G = granulocytes; ROS = Reactive Oxygen Species; TAC = Total Antioxidant Capacity; MDA = MalonDiAldehyde; Lipoperox = lipoperoxidation; RFU = Relative Fluorescence Unit. Data are reported as geometric mean and (range). General linear model adjusted for age, gender, number of medications, adherence score to MDLDL: Low-Density Lipoprotein; HDL: High-Density Lipoprotein. * *p* < 0.05 for comparison between pre- and post-intervention values (paired t-test). ^a^ Comparison between absolute changes induced by the two interventions (General linear model for repeated measurements).

**Table 4:** Modifications in the inflammatory profile ^a^.

Variable	Khorasan Pre	Khorasan Post	Control Pre	Control Post	Change, Khorasan	Change, Control	*p*
**Interleukin-1ra**, pg mL^−1^	38.2 (11.2–65.5)	30.4 (15.1–45.7)	40.8 (26.6–55)	36.8 (24.8–48.8)	−7.8 (−36.5; 20.9)	−4 (−15.7; 7.6)	0.5
**Interleukin-4**, pg mL^−1^	0.48 (0.27–0.68)	0.44 (0.16–0.72)	0.48 (0.21–0.76)	0.51 (0.26–0.76)	−0.04 (−0.3; 0.2)	0.03 (−0.4; 0.5)	0.1
**Interleukin-6**, pg mL^−1^	2.26 (1.50–3.03)	1.53 (1.16–1.90)	3.16 (1.51–4.81)	3.30 (1.24–6.37)	−0.73 (−1.6; 0.1)	0.14 (−3.3; 3.6)	0.9
**Interleukin-8**, pg mL^−1^	10.9 (6.2–15.5)	5.6 (3.2–8)	8.1 (6.2–10)	12.7 (3.3–22.1)	−5.3 (−11.1; 0.5)	4.6 (−5; 14.3)	0.4
**Interleukin-10**, pg mL^−1^	11.6 (4.9–18.3)	9.6 (5.5–13.7)	10 (7–12.9)	13.6 (2.9–29.9)	−2 (−7.8; 3.9)	3.6 (−3.2; 20.4)	0.3
**Interleukin-12**, pg mL^−1^	30.8 (14.3–47.3)	24.2 (15.6–32.8)	22.2 (13.3–31.2)	23.8 (11.9–35.6)	−6.6 (−18.8; 5.6)	1.6 (−9.4; 12.5)	0.6
**Interleukin-17**, pg mL^−1^	4.2 (1.3–7)	8.4 (4.9–11.8)	8.6 (2.6–14.6)	6.6 (3.2–10)	4.2 (−1.7; 10.1)	−2 (−8.3; 4.3)	0.4
**INF-gamma**, pg mL^−1^	21.6 (12.8–30.5)	13.8 (9.2–18.5)	23.2 (15.9–30.7)	19.2 (10.9–27.5)	−7.8 (−16; 0.4)	−4 (−15.8; 7.6)	0.5
**MCP-1**, pg mL^−1^	43.8 (31.6–56.1)	72.2 (50.6–93.7)	61.9 (39.6–84.4)	47.9 (30.1–65.8)	−8.1 (−30.7; 14.5)	−14 (−33.8; 5.7)	0.3
**MIP-1beta**, pg mL^−1^	80.3 (59–101.6)	72.2 (50.6–93.7)	88.7 (61.1–108.4)	73 (54.3–91.6)	−8.1 (−30.7; 14.5)	−15.8 (−33.9; 2.4)	0.7
**TNF-alpha**, pg mL^−1^	8.3 (3.9–12.6)	3.9 (1.4–6.4) *	6.5 (2.9–9.9)	4.6 (0.9–8.2)	−4.4 (-8.7; −0.007)	−1.9 (−5.5; 1.7)	0.04
**VEGF**, pg mL^−1^	170.4 (90–250.9)	124.1 (64.7–183.6)	137.6 (74.9–200.2)	93.2 (44.9–142.4)	−46.3 (−97; 4.4)	−44.4 (−97.1; 8.2)	0.9

INF-gamma = Interferon-gamma; MCP-1 = Monocyte Chemotactic Protein-1; MIP-1beta = Macrophage Inflammatory Protein-1 beta; TNF-alpha = Tumour Necrosis Factor-alpha; VEGF = Vascular Endothelial Growth Factor. Data are reported as geometric mean and (range). General linear model adjusted for age, gender, number of medications, adherence score to MD. * *p* < 0.05 for comparison between pre- and post-intervention values (paired *t*-test). ^a^ Comparison between absolute changes induced by the two interventions (General linear model for repeated measurements).

Interestingly, blood glucose and insulin levels were also shown to decrease significantly after the ancient organic khorasan wheat replacement diet, but not after the control. Although baseline blood glucose levels were within the recommended target level for patients in secondary cardiovascular protection, levels were, however, significantly higher than those reported for the healthy population in our previous study [9]. It has been reported that blood glucose, even within window of the normal range, is a strong independent predictor of cardiovascular mortality in non-diabetic patients with CVD [18]. Similarly, baseline insulin levels were significantly higher in comparison to baseline levels of the healthy population [9]. Down-regulation of insulin levels is also important in reducing cardiovascular risk as insulin may signal the perpetuation of pro-inflammatory cytokines [2]. Hence, the effect of organic khorasan wheat on both of these parameters is noteworthy, as is the increase in magnesium content. Lower serum magnesium levels are positively associated with higher risks of coronary disease [17]. Moreover, both magnesium and vanadium have been shown to lower plasma glucose, and the therapeutic potential of both elements in the prevention of diabetes has been demonstrated [17,19]. Grains represent a valuable source of vanadium, and the present results show significantly higher vanadium content in khorasan wheat flour than in the control wheat flour, with the same reduction in glucose noted in our previous study [9].

The present study was subject to limitations in the study design. The number of participants (22 in total) represents a main limitation of the present study. Although the results are promising, these findings need to be interpreted with caution unless validated in a bigger sample population. Another main limitation is the lack of a complete evaluation of dietary profile in the two groups of interventions. We have performed a simple and easy questionnaire evaluating the adherence to the Mediterranean diet, but this suffers from many drawbacks, giving us little information on nutritional profile of subjects in the two intervention phases. A food-frequency questionnaire would have been the better choice to investigate possible differences between the two interventions. Moreover, they reported no changes in smoking habit and medication to control ACS related and additional risk factors.

## 5. Conclusions

In conclusion, results demonstrate that an organic khorasan wheat replacement diet is effective at significantly reducing levels of total cholesterol, LDL-C, blood glucose, insulin, monocyte and lymphocyte ROS production and lipoperoxidation, and TNF-alpha, and increasing magnesium content in patients with ACS. Despite stringent medicinal therapies, the present study shows that the ancient organic khorasan wheat has an additive protective effect by further down-regulating several key parameters/markers, including those considered important for secondary prevention of ACS. This protection is not afforded by conventional control wheat.

## References

[1] Morrow D.A. (2010). Cardiovascular risk prediction in patients with stable and unstable coronary heart disease. Circulation.

[2] De Caterina R., Zampolli A., Turco S., Madonna R., Massaro M. (2006). Nutritional mechanisms that influence cardiovascular disease. Am. J. Clin. Nutr..

[3] Sofi F., Macchi C., Abbate R., Gensini G.F., Casini A. (2014). Mediterranean diet and health status: Un updated meta-analysis and a proposal for a literature-based adherence score. Public Health Nutr..

[4] Yusuf S., Hawken S., Ounpuu S., Dans T., Avezum A., Lanas F., McQueen M., Budaj A., Pais P., Varigos J. (2004). Effect of potentially modifiable risk factors associated with myocardial infarction in 52 countries (the INTERHEART study): Case-control study. Lancet.

[5] Dehghan M., Mente A., Teo K.K., Gao P., Sleight P., Dagenais G., Avezum A., Probstfield J.L., Dans T., Yusuf S. (2012). Relationship between healthy diet and risk of cardiovascular disease among patients on drug therapies for secondary prevention: A prospective cohort study of 3,1546 high-risk individuals from 40 countries. Circulation.

[6] Dinelli G., Marotti I., Bosi S., Benedettelli S., Ghiselli L., Cortacero-Ramírez S., Carrasco-Pancorbo A., Segura-Carretero A., Fernández-Gutiérrez A. (2007). Lignan profile in seeds of modern and old Italian soft wheat (*Triticum aestivum* L.) cultivars as revealed by CE-MS analyses. Electrophoresis.

[7] Dinelli G., Segura-Carretero A., di Silvestro R., Marotti I., Fu S., Benedettelli S., Ghiselli L., Fernandez-Gutierrez A. (2009). Determination of phenolic compounds in modern and old varieties of durum wheat using liquid chromatography coupled with time-of-flight mass spectrometry. J. Chromatogr. A.

[8] Sofi F., Ghiselli L., Cesari F., Gori A.M., Mannini L., Casini A., Vazzana C., Vecchio V., Gensini G.F., Abbate R. (2010). Effects of short-term consumption of bread obtained by an old Italian grain variety on lipid, inflammatory, and hemorheological variables: An intervention study. J. Med. Food.

[9] Sofi F., Whittaker A., Cesari F., Gori A.M., Fiorillo C., Becatti M., Marotti I., Dinelli G., Casini A., Abbate R. (2013). Characterization of Khorasan wheat (Kamut) and impact of a replacement diet on cardiovascular risk factors: Cross-over dietary intervention study. Eur. J. Clin. Nutr..

[10] Becatti M., Fiorillo C., Gori A.M., Marcucci R., Paniccia R., Giusti B., Violi F., Pignatelli P., Gensini G.F., Abbate R. (2013). Platelet and leukocyte ROS production and lipoperoxidation are associated with high platelet reactivity in Non-ST elevation myocardial infarction (NSTEMI) patients on dual antiplatelet treatment. Atherosclerosis.

[11] Autieri M.V. (2012). Pro- and Anti-Inflammatory cytokine networks in atherosclerosis. ISRN Vasc. Med..

[12] Erickson K.L., Medina E.A., Hubbard N.E. (2000). Micronutrients and innate immunity. J. Infect. Dis..

[13] Quiñones M., Miguel M., Aleixandre M. (2013). Beneficial effects of polyphenols on cardiovascular disease. Pharm. Res..

[14] Lee A.S., Kim J.S., Lee Y.J., Kang D.G., Lee H.S. (2012). Anti-TNF-α Activity of *Portulaca oleracea* in Vascular Endothelial Cells. Int. J. Mol. Sci..

[15] Ridker P.M., Rifai N., Pfeffer M., Sacks F., Lepage S., Braunwald E. (2000). Elevation of Tumor Necrosis Factor-α and increased risk of recurrent coronary events after myocardial infarction. Circulation.

[16] Geisler T., Bhatt D.L. (2004). The role of inflammation in atherothrombosis: Current and future strategies of medical treatment. Med. Sci. Monit..

[17] Fardet A. (2010). New hypotheses for the health protective mechanisms of whole-grain cereals: What is beyond the fibre?. Nutr. Res. Rev..

[18] Port S.C., Goodarzi M.O., Boyle N.G., Jennrich R.I. (2005). Blood glucose: A strong risk factor for mortality in nondiabetic patients with cardiovascular disease. Am. Heart J..

[19] Srivastava A.K., Mehdi M.Z. (2005). Insulino-mimetic and anti-diabetic effects of vanadium compounds. Diabet Med..

